# Gb3/cd77 Is a Predictive Marker and Promising Therapeutic Target for Head and Neck Cancer

**DOI:** 10.3390/biomedicines10040732

**Published:** 2022-03-22

**Authors:** Lorena García-Hevia, Débora Muñoz-Guerra, Íñigo Casafont, Carmelo Morales-Angulo, Victor J. Ovejero, David Lobo, Mónica L. Fanarraga

**Affiliations:** 1 The Nanomedicine Group, Institute Valdecilla-IDIVAL, 39011 Santander, Spain; dmunoz@idival.org (D.M.-G.); casafonti@unican.es (Í.C.); vovejerohcas@msn.com (V.J.O.); dlobo28@gmail.com (D.L.); fanarrag@unican.es (M.L.F.); 2 Molecular Biology Department, Faculty of Medicine, Universidad de Cantabria, 39011 Santander, Spain; 3 Anatomy & Cell Biology Department, Faculty of Medicine, Universidad de Cantabria, 39011 Santander, Spain; 4 Servicio de Otorrinolaringología, Hospital Universitario Marqués de Valdecilla, 39011 Santander, Spain; carmelo.morales@unican.es; 5 Departamento de Cirugía General y Digestiva, Hospital Universitario Marqués de Valdecilla, 39011 Santander, Spain; 6 ENT Department, Hospital Universitario Marqués de Valdecilla, 39011 Santander, Spain

**Keywords:** ganglioside, cancer, resistance, squamous cell carcinoma

## Abstract

Head and neck squamous cell carcinoma is the sixth leading cancer in the world. This cancer is difficult to treat and is characterized by recurrences that are often fatal. This cancer is generally removed surgically, but it often regrows from the edges of the lesion from where most recurrences reappear. In this study, we have investigated if the expression of GB3 in human cell lines, tissues from patient biopsies, and a murine animal model could be used as an early and determinant marker of HNC. We found that in all the investigated systems, this marker appears in neoplastic cells from the very early stages of their malignant transformation. Our conclusions support the hypothesis that GB3 is a reliable and independent target for HNC identification and selective delivery of treatments. Furthermore, we show that the level of expression of this marker correlates with the degree of malignancy of the tumor. These studies suggest that GB3 may provide the basis for the early identification and new targeted therapies for head and neck cancer.

## 1. Introduction

Head and neck carcinoma (HNC)**,** and more particularly the squamous cell carcinoma of the head and neck (HNSCC), is the sixth most common cancer worldwide [1,2]. This malignancy includes a great heterogeneity of tumors that arise from the mucosal surfaces of the oral cavity, oropharynx, larynx, nasal cavity, or salivary glands. The main risk factors are tobacco and alcohol [3,4,5], but also the human papillomavirus infection [6,7]. Other factors that also contribute to development of this cancer are poor oral hygiene, opium consumption, [8], immunodeficiency [9], occupational exposure [10], diet [11], radiation [12] or genetic factors [13,14].

When diagnosed at stage I or II, HNSCC can be cured with surgery and/or radiation therapy. Unfortunately, up to 75% of patients are diagnosed with locally advanced diseases. In these more advanced stages, survival rates are on average 5 years after diagnosis (40–50%), and produce 370,000 deaths each year [15,16,17]. This results from (i) poor detection of the affected preneoplastic areas, (ii) poor clearance of affected tissue, (iii) poor penetration of the systemic drug into the tumor mass, and/or (iv) surgical complexity since the vital importance of the anatomical structures adjacent to the tumor can limit the resection. The heterogeneity of the affected tissues and also the fact that it is difficult to determine the edges of the lesion upon tumor surgical removal lead to more than 65% of local recurrences, many of which are fatal despite treatment with radiation therapy or chemotherapy [18]. Hence, HNSCC tumors represent an oncological challenge, both due to their incidence and mortality, as well as the functional and aesthetic morbidity they cause.

In this context, there is an urgent need for the development of new drugs or therapeutic options to be applied in refractory advanced diseases. The greatest efforts are directed to the search for drugs that target the epidermal growth factor receptor (EGF-R). In this sense, there exist several anti-EGFR agents based on tyrosine kinase inhibitors binding to the intracellular kinase domain of EGFR such as gefitinib, erlotinib or dacomitinib, or dual inhibitors of EGFR and HER2, such as lapatinib or afatinib, which are currently under investigation in phase II and III clinical trials [19,20,21,22]. In addition, Cetuximab is a monoclonal antibody that binds to the extracellular domain of EGF-R and is used in unresectable HNSCC or as an adjuvant to radiation therapy in resectable tumors [23,24]. Unfortunately, although the large number of patients benefitting from disease stabilization with Cetuximab is significant, the response rate to the antibody falls after approximately 4 months, as resistance develops during this period [25]. However, despite the fact that treatment with Cetuximab as monotherapy does not appear to offer encouraging results, the addition of Cetuximab to radiotherapy reduces the risk of locoregional failure by 32% and a 26% risk of death, resulting in a nearly 20-month increase in median survival [23]. Treatment with Cetuximab shows, on the one hand, the positive effects that targeted therapies can produce in cancer. However, on the other hand, it also shows the enormous plasticity that neoplastic cells have to generate resistance and bypass targeted treatments [17]. 

In the search for specific targeting receptors on the surface of HNSCC cells, we have looked for aberrant glycosylation patterns specific to this neoplasm. Glycans are involved in many molecular and cell biology processes occurring in cancer. These include cell signaling and communication, tumor cell invasion, cell–matrix interactions, neo-angiogenesis, immune modulation, and metastasis formation [26]. The levels of glycosylation and changes in glycosyl structure are closely linked to cancer progression and malignant transformation. Indeed, changes in surface glycans are a hallmark of different cancers [26,27]. Although the glycobiology of cancer in general, and more particularly in HNSCC, is still a new field that needs to be deeply studied, changes in glycosylation patterns have been associated with malignancy and drug resistance in HNSCC [28,29] and provide potential strategies to identify and control cancer stem cell expansion, epithelial–mesenchymal transition, tumor-related immunity escape, and autophagy. Indeed, glycoproteins with altered glycosylation can be used as biomarkers for the early diagnosis, monitoring, and prognostication of HNSCC [30]. 

Recently, we have found that the glycosphingolipid globotriaosylceramide (GB3 or CD77), a receptor on the surface of many neoplasms of the digestive tract, is also a marker for preneoplastic and malignant HNC cells [31,32]. More interestingly, this receptor has an added value since it specifically binds to the Shiga toxin, which is a high-affinity innocuous natural ligand that, upon binding to the receptor, triggers retrograde receptor internalization through the Golgi network to the endoplasmic reticulum [33,34,35,36,37,38]. Several studies have shown how the toxin cell invasion mechanism can be used to introduce peptides or diagnostic/therapeutic nanomaterials [39] inside cells. Hence, GB3 overexpression in localized and accessible cancer, such as HNSC, offers new exciting possibilities for preneoplastic and malignant specific cell identification—i.e., during surgery to remove surgical borders of the lesion, or in designing targeted therapies based on nanoparticles, both to deliver drugs straight inside the cancer cells or to place the nanomaterials in the cellular realm [39]. This would allow interfering with natural cellular processes; for instance, carbon nanotubes can interfere with cell division and microtubule dynamics [40,41,42,43], and magnetic or gold nanoparticles can be used to trigger hyperthermia to destroy GB3 positive cancer cells from inside out [39].

In summary, the importance of this marker as a system for the early detection of tissues affected by HNSCC, and as a mechanism to specifically introduce encapsulated therapies intracellularly, makes the determination of the distribution of GB3 in HNSCC of great interest. Here, we qualitatively and quantitatively describe the expression of GB3 in human head and neck cancer cell lines, mouse HNSCC models, and in clinically well-characterized patients with well-diagnosed different grades of HNC with the hope that this ligand can be used in the short–medium term to selectively identify HNSCC cells and selectively drive cytotoxic therapies. This would allow locally boosting the chemotherapy impact, making the treatments more effective and less toxic. 

## 2. Materials and Methods

### 2.1. Cell Culture

Detroit 562 (human pharynx epithelial carcinoma cells obtained from ATCC (Manassas, VA, USA) Ref CCL-138), HaCat cells (kindly provided by Dr. Quintanilla Lab), and MCF7 cells (human breast cancer) were used for the study. All of them were grown in Iscove’s Modified Dulbecco’s Medium containing 10% bovine serum (Thermo, Waltham, MA, USA). Cells were grown under standard conditions (37 °C, 5% CO_2_).

### 2.2. Immunofluorescence

Cells were fixed with 4% paraformaldehyde and stained with Hoechst 33258 (Sigma-Aldrich^®^, St. Louis, MO, USA). Alexa647-anti-Human CD77 (BD Pharmingen™, Wokingham, UK) was used for immunostaining the GB3 receptor. Confocal images were taken with a Nikon A1R microscope. All fluorescent images are pseudo-colored.

### 2.3. Flow Cytometry

A total of 10^4^ Detroit, HaCat, and MCF-7 cells were immunostaining with Alexa647-anti-Human CD77 for 30 min and then fixed in 4% paraformaldehyde. Cells were analyzed using cytoFLEX equipment and CytExpert software (Beckman Coulter, Brea, CA, USA). Three experimental replicas were performed per condition (Supplementary Materials Figure S1).

### 2.4. Preclinical Murine Models

Ethical permissions for this study were approved and obtained from the Bioethics Committee University of Cantabria, General Directorate of Livestock, Government of Cantabria (Project Ref. PI-11-21, approved 25 November 21). 

C57BL/6 mice (12 weeks old) were obtained from and raised at the Experimentation Service (SEEA) of the University of Cantabria. Animals were maintained, handled, and sacrificed following the directive 2010/63/UE. Mice were treated with 100 μg/mL of 4-Nitroquinoline-1-oxide (4NQO, Sigma Aldrich, St. Louis, MO, USA) in drinking water for 16 weeks and then followed for 4 weeks (water was changed once a week), as previously defined [44,45,46]. After 16 weeks of carcinogen treatment, mice were sacrificed bi-weekly and oral lesions were assessed. Dissected tissues were fixed in 10% formalin, embedded in paraffin, sectioned, deparaffinized, and stained with hematoxylin-eosin (Sigma Aldrich, St. Louis, MO, USA).

### 2.5. Frozen Animal Tissues

The tongues of euthanized mice were dissected and photographed. Fixed tissues were embedded in sucrose and frozen in optimal cutting temperature compound (OCT, Tissue Tek, Alphen aan den Rijn, The Netherlands) for 6 μm with cryostat sectioning. The sections were immunostained Alexa647-anti-CD77 and stained with Hoechst (Sigma Aldrich, St. Louis, MO, USA) or deparaffinized and stained with hematoxylin-eosin.

### 2.6. Human Biopsy Samples

Samples and data of human squamous cell carcinoma from the donors included in this study were provided by two national biobanks integrated into the Spanish National Biobanks Network, the Principado de Asturias BioBank (PT17/0015/0023) and the Valdecilla Hospital Biobank (PT20/00067). Samples were processed following standard operating procedures with the appropriate approval of the Ethical and Scientific Committee (Project Ref. PI19/00349). Seventeen different biopsy samples and their corresponding healthy tissue sections were deparaffinized with decreasing concentrations of ethanol, blocked with albumin, and then immunostained with Alexa647-anti-CD77 and stained with Hoechst or treated with hematoxylin-eosin. These hematoxylin-eosin samples were photographed under the stereo microscope ZEISS Stemi 508.

### 2.7. Quantitative Examination of GB3 Expression

The human samples immunostained with Alexa647-anti-CD77 were photographed with a Nikon A1R Confocal microscope. These images were processed with the ImageJ software. The results are shown as percentages of the total area of the sample (Figure S4).

### 2.8. Statistical Analysis

The GB3 expression in human HNC tissues was evaluated using one-way analysis of variance (ANOVA) statistical analysis (SPSS software, version 19.0, SPSS Inc., Chicago, IL, USA). Statistical significance was assumed at *p* < 0.05, and pairwise comparisons were conducted when ANOVA showed significant results. All quantitative results are expressed as mean values with their corresponding standard deviation error bars. The total number of events and the confidence levels obtained in the experiment (*n*) are all indicated in the figure caption.

## 3. Results

### 3.1. Detection of GB3 in Preneoplastic and Malignant Head and Neck Cells

First, GB3 expression was confirmed in cell cultures of a head and neck cell line. We chose Detroit 562 as a representative malignant HNC cell line [47]. These cells were derived from a metastatic pleural effusion of a human pharynx epithelial carcinoma. Human epidermal keratinocyte line HaCaT cells were used as a preneoplastic cell model. Finally, as a negative control cell line, we chose human breast cancer MCF7 cells, which have been previously demonstrated to be GB3-negative. 

**Figure 1:**
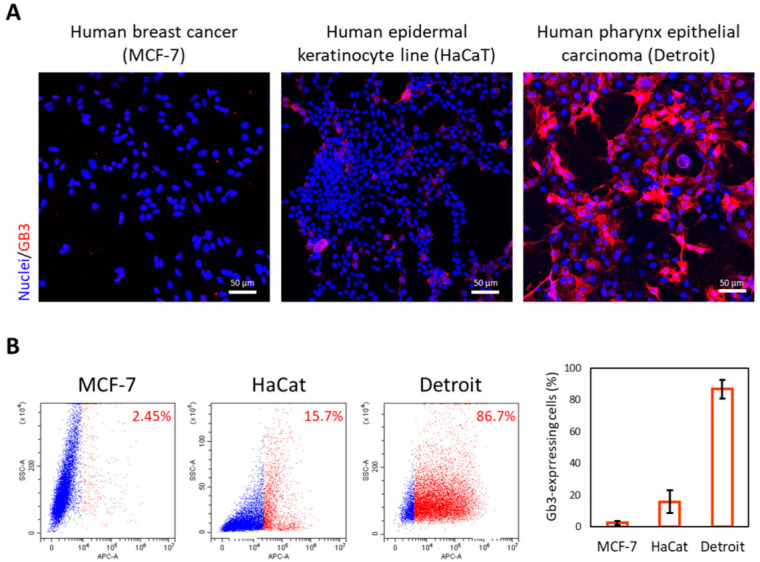
Detection of GB3 in human cell lines. (**A**) Confocal microscopy projection images of live human cell cultures immunostained with anti-CD77 recognizing GB3 (red channel). Cell nuclei are stained with Hoechst (blue channel). Scale bar = 50 μm. (**B**) Flow cytometry analysis of these cultures confirmed that 86% of the malignant HNC neoplastic cells (Detroit 562), 16% of precancerous keratinocytes (HaCat), and 2.4% breast cancer cells (MCF7) presented detectable GB3 levels. The histogram shows the average of three experimental replicas where ca. 10^6^ cells were analyzed per study (see Figure S1).

As a preliminary approach, we performed immunofluorescence with an anti-GB3 antibody in live cell cultures. Cells were then fixed and imaged using confocal microscopy. Figure 1A shows negative MCF7 cells (negative control) compared to the preneoplastic (HaCaT) and malignant cells (Detroit 562) that were both positive. However, this latter cell line was significantly more positive than the human keratinocytes. 

To verify and quantify GB3 expression in the three cell lines, we next used flow cytometry. Figure 1B shows how 84% of the malignant neoplastic cells (Detroit 562) presented GB3 on their surfaces compared with nearly 16% of the human keratinocytes (HaCat). On the contrary, less than 3% of the human breast cancer cells (MCF7) presented detectable GB3 levels. 

In summary, these studies demonstrate GB3 expression in the surface of malignant HNC cells by two complementary methodologies. Furthermore, this study suggests the amount of detectable GB3 is directly proportional to the degree of malignancy of the cells. Hence, these studies suggest that GB3 could be a valid marker to identify tissues affected by this neoplasia. 

### 3.2. Gb3 Expression in an Hnc Mouse Model

To corroborate these in vitro results in vivo, we used an HNC animal model. To do this, we reproduced a well-established squamous cell carcinoma murine model which has been validated in several studies using the carcinogen 4-NQO (Materials and Methods) [44,45,46]. This treatment causes HNSSC lesions in the oral cavities of mice, particularly in the tongues and esophagus [46]. Tissue from the tongue and esophagus of the treated mice and untreated controls were dissected, fixed, embedded, and sectioned. Hyperplasia of the tongue and esophagus squamous epithelium was corroborated upon histopathological evaluation of hematoxylin and eosin-stained sections (Figure S2).

**Figure 2:**
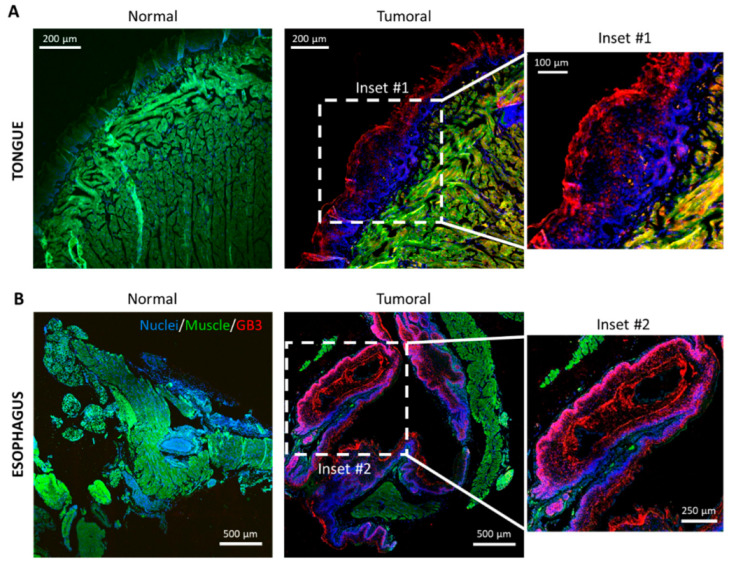
GB3 expression in an HNC mouse model. Multichannel confocal microscopy images of sections of murine (**A**) tongues and (**B**) esophagus. Sections were immunostained with Alexa647-anti-CD77 (red channel). Nuclei were stained with Dapi (blue channel) and the autofluorescence of the muscle fibers appear in the green channel. The red color is indicative of GB3 expression. Scale bars = 500, 250 μm.

The expression of GB3 was analyzed in these sections by immunohistochemistry with an Alexa647-anti-CD77 antibody. Figure 2 shows representative confocal microscopy images of the murine tissues. Immunostained sections revealed how the epithelial hyperplasia was accompanied by conspicuous GB3 immunostaining. Both the tongue (Figure 1A) and esophagus (Figure 1B) of treated mice displayed zones with strong GB3 reactivity (Insets #1 and #2, respectively) compared to the untreated controls. 

Hence, GB3 expression in animal models confirms results observed in human cell lines, validating the quantitative and qualitative results obtained in vitro.

### 3.3. Overexpression of GB3 in Biopsy Samples of Human HNC Tissues

GB3 expression was next validated retrospectively on clinical samples of several human squamous cell carcinoma of the head and neck tissues obtained from the local human tissue sample biobank (See Materials and Methods). These tissue sections are representative biopsy samples from patients who were diagnosed and clinically classified as indicated in the figure. These tissue sections proceeded from cases cataloged with different degrees of malignancies. 

**Figure 3:**
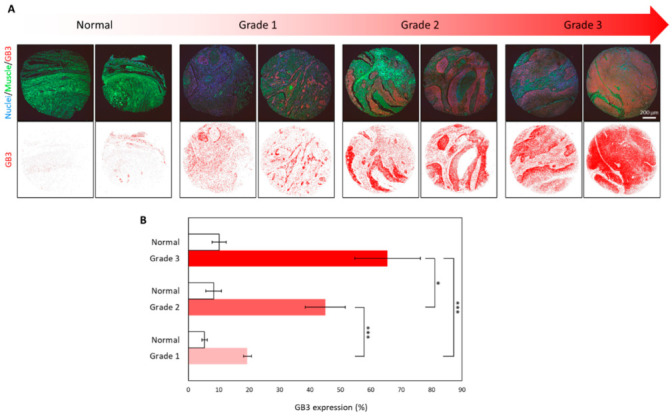
GB3 expression in sections of paraffin-embedded biopsy samples. Confocal microscopy images of tissue samples from patients displaying different clinical grades of HNSCC. (**A**) Top panels: Multichannel confocal microscopy images on tissues immunostained with Alexa647-anti-CD77 (red-channel). Nuclei were stained with Dapi (blue-channel), and tissue autofluorescence appears in the green channel. GB3 immunohistochemistry appears in the red channel. Normal controls and the different anatomopathological grades of HNC malignancy are indicated. The bottom panel shows the GB3 signal digitally extracted from the confocal images. The red color in the images corresponds to the GB3 immunostaining signals detected. Scale bars = 200 μm. (**B**) Quantitative examination of GB3 expression (*n* = 33, *p* = 0, 6.3 × 10^−7^ and 0.019 comparing grade 1 to grade 2, grade 1 to grade 3 and grade 2 to grade 3, respectively; *** *p* < 0.001 and * *p* < 0.010).

For the study, we used a tissue array of three different samples of a total of 17 patients. Healthy sample tissue from the same patients was processed in side-by-side sections of the array to be directly comparable. All these biopsy samples were processed simultaneously and were immunostained on the same slide to prevent possible artifacts. Hematoxylin/eosin staining was used to evaluate the biopsy samples histopathologically and to confirm the abnormal growth of epithelial cells and the dysplasia related to the degree of malignancy (Figure S3). Tissue sections were immunostained with the Alexa647-anti- CD77 antibody as before. To evaluate GB3 expression, we used confocal microscopy imaging. To perform a semi-quantitative evaluation of the expression of GB3, all images were captured using identical confocal settings (identical HV, offset, and laser power). The semi-quantification of GB3 expression in the tissues was performed using software imaging tools. To do this, the distribution of the fluorescent signal relative to the total area of the images of the immunostained section (Figure 3A, bottom) was taken into account. 

Figure 3B shows single Z confocal microscopy multichannel images taken from samples of patients diagnosed with different HNSCC grades. Tissues obtained from grade 1 malignancies presented ca. 20% of GB3 immunoreactivity. Patients diagnosed with grade 2 exhibited ca. 45% GB3 immunoreactivity in their samples. Finally, grade 3 sample tissues showed ca. 70% GB3 immunostaining. On the contrary, using an identical imaging setup, we detected no GB3 immunostaining in normal tissue samples (Figure 3B).

This experiment performed on real clinical samples significantly supports the hypothesis that malignant transformation of HNC cells is accompanied by changes in the expression of this GB3. The strong correlation observed suggests that this marker could be criterial to identify and treat HNSCC.

## 4. Discussion

HNC are a heterogeneous group of tumors that, in general, are biologically aggressive and include the following subsites: oral cavity, nasopharynx, oropharynx, hypopharynx, larynx, paranasal sinuses, nasal cavity, and salivary glands. HNC, accounting for ~4% of all malignancies worldwide and 5% of the mortality of all cancers, could significantly benefit from early diagnosis and detection since the average survival of patients is 40–50% five years after diagnosis. Approximately two-thirds of HNC patients are diagnosed in advanced stages when cancer evolution is invariably fatal. Late diagnosis requires radical treatment and multimodality therapy with reduced cosmetic compromise. Additionally, despite advances in surgery and radiotherapy, up to 50% of locally advanced tumors fatally relapse, usually within the first 2 years after treatment, with limited options for salvage surgery or reirradiation. On the contrary, patients detected at the early stage of disease present cure rates of 70–90% five years after diagnosis. 

Early diagnosis/screening of cancer saves lives and is an important public health strategy. So, given the increasing number of cancer diagnoses expected in the coming years, cancer screening programs are necessary. Unfortunately, to date, no specific pathognomonic cancer marker allows a global and systematic HNC screening. Thus, finding tumor biomarkers is crucial for the diagnostic, evaluation, and monitoring of the course of neoplasias while helping to predict response to treatment. The FDA has approved around 30 biomarkers after robust in vitro tests [48]. However, the HNSCC biomarkers described so far have not been validated by the FDA for clinical uses since they show low specificity and clinical relevance, high turnaround time, and high costs [49]. Instead, time-consuming laboratory methods such as gene panel sequencing on tissue or liquid biopsy samples, or methods such as X-ray, CT scan, and MRI and PET scan, are the alternative. However, these expensive and sophisticated diagnostic systems are only occasionally used for diagnostic confirmation. So, there is an urgent need for HNC predictive biomarkers for HNC screening.

In the search for specific molecules to identify HNC, we have looked for aberrant glycosylation patterns specific to this neoplasm. Here, we demonstrate the expression of GB3 in HNSCC cells and tissues. Our experiments show that GB3 immunostaining directly compares with the malignant transformation of the cells and tissues. The results demonstrate a direct correlation of the clinical histopathological degrees with the immunostaining levels in two ways: intensity and surface signal. This way, human HNSCC tissue of grade 1 has 15% more GB3 than normal tissue, which expresses insignificant basal GB3 levels. Furthermore, grade 2 HNC shows a 40%, and grade 3 over a 60% increment in GB3 immunostaining compared to the basal levels. In agreement with these results, previous studies showed that dedifferentiated ovarian carcinomas [50] and breast and colorectal cancer had high GB3 levels [51,52] and drug-resistant tumors compared to the primary tumor [29]. In addition, a correlation between tumor progression and GB3 expression was also observed [52]. We believe these results may be crucial to develop new therapies specifically targeting head and neck cancer where the mortality rate associated with this regrowth of this cancer is very high. Moreover, this marker might also serve for early diagnoses or to identify the edge of the lesion. Immunostaining with GB3 could serve as a way to identify the tumoral edges and prevent regrowth, improving treatments with systemic chemotherapy. 

**Figure 4:**
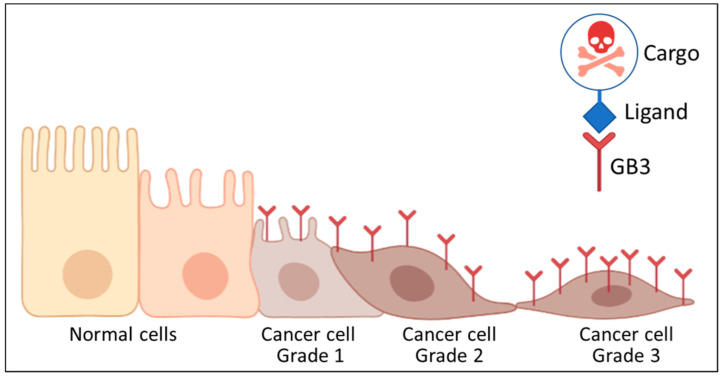
Schematic representation of the transformation process and different GB3 levels of expression. The GB3 is a possible receptor to target diagnostic or therapeutic solutions into malignant cells. Created with Biorender.com.

Interestingly, since GB3 is the receptor of well-known ligands such as the Shiga toxin, the ligand domain of the protein might also be used to develop targeted therapies to treat HNC cells and tissues specifically (Figure 4), for example, to introduce chemotherapy-loaded nanosystems into GB3-positive cells. Alternatively, nanosystems that activate by light or magnetism to produce focal hyperthermia—such as carbon nanotubes, gold, or magnetic nanoparticles—can be targeted to these cells to boost treatment with traditional therapies. In addition, GB3 can also be used as a targeting/therapeutic strategy in the treatment of many other cancers where this sugar is overexpressed. These include pancreatic cancer [53], colon cancer and metastasis [52,53], breast [54] and ovarian cancer [52], or astrocytomas and neuroblastomas [55]. The huge potential of this HNC marker continues to be investigated.

## 5. Conclusions

GB3 or globotriaosylceramide is detected in HNSCC cells and tissues in different model systems. Its expression correlates with the degree of cell/tissue malignancy. This study proposes this marker as a candidate for early diagnosis and evaluation of the degree of these head and neck cancers, and to direct therapies specifically to the interior of the transformed cells to improve the effects of chemotherapy locally, reducing systemic toxicity.

## Data Availability

The data presented in this study are available on request from the corresponding author.

## Supplementary Materials

The following supporting information can be downloaded at: https://www.mdpi.com/article/10.3390/biomedicines10040732/s1, Figure S1: Flow cytometry experiments, Figure S2: Hematoxylin and eosin-stained sections of HNC mouse, tongue and esophagus, Figure S3: Hematoxylin and eosin-stained HNC human tissue sections and Figure S4: GB3 quantification in human samples.
