# Supplementary material for: Gb3/cd77 Is a Predictive Marker and Promising Therapeutic Target for Head and Neck Cancer
Source: Biomedicines. 2022 Mar 22;10(4):732. doi: 10.3390/biomedicines10040732 (PMC9029501; doi:10.3390/biomedicines10040732)
Supplement: Supplementary file 1 [file biomedicines-10-00732-s001.zip › biomedicines-1620780-supplementary.pdf]

Suplementary Information

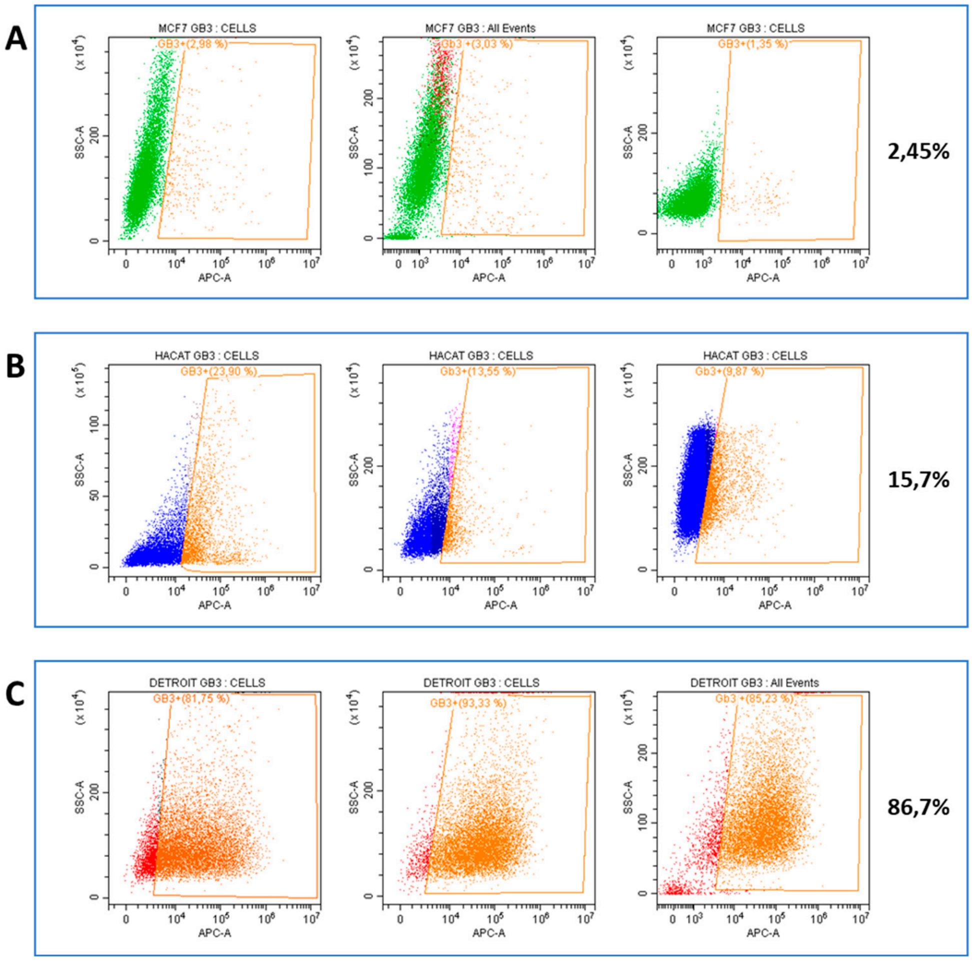

Figure S1. Flow cytometry experiments of (**A**) breast cancer cells (MCF7), (**B**) precancerous keratinocytes (HaCat) and (**C**) malignant HNC neoplastic cells (Detroit 562), immunostained with Alexa647-anti-CD77 for the GB3 expression evaluation. These replicas per condition have been used to create the graph of Figure 1B.

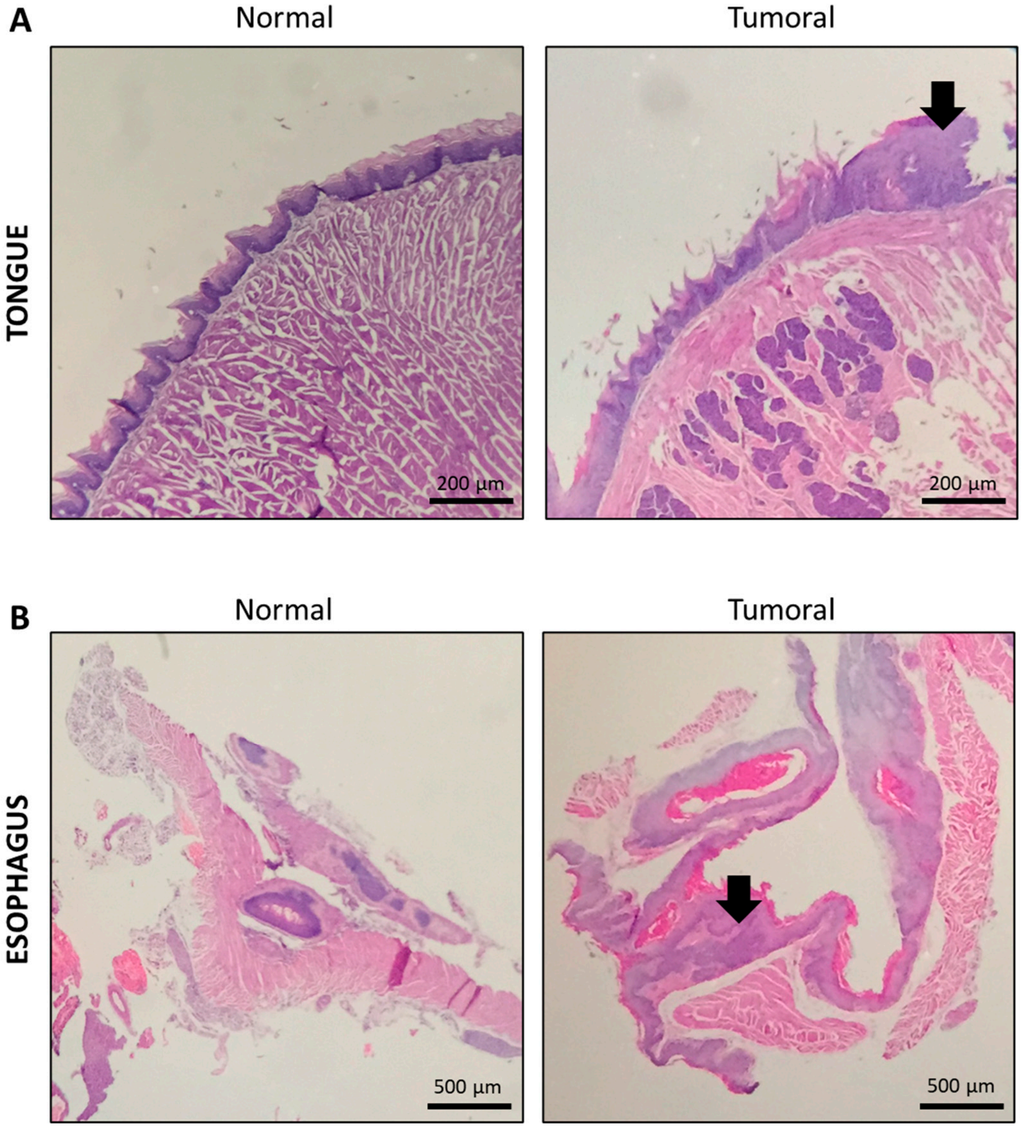

Figure S2. Hematoxylin and eosin-stained sections of HNC mouse (**A**) tongue and (**B**) esophagus. Both tumoral tissues show remarkable hyperplasia in the squamous epithelium (black arrows).

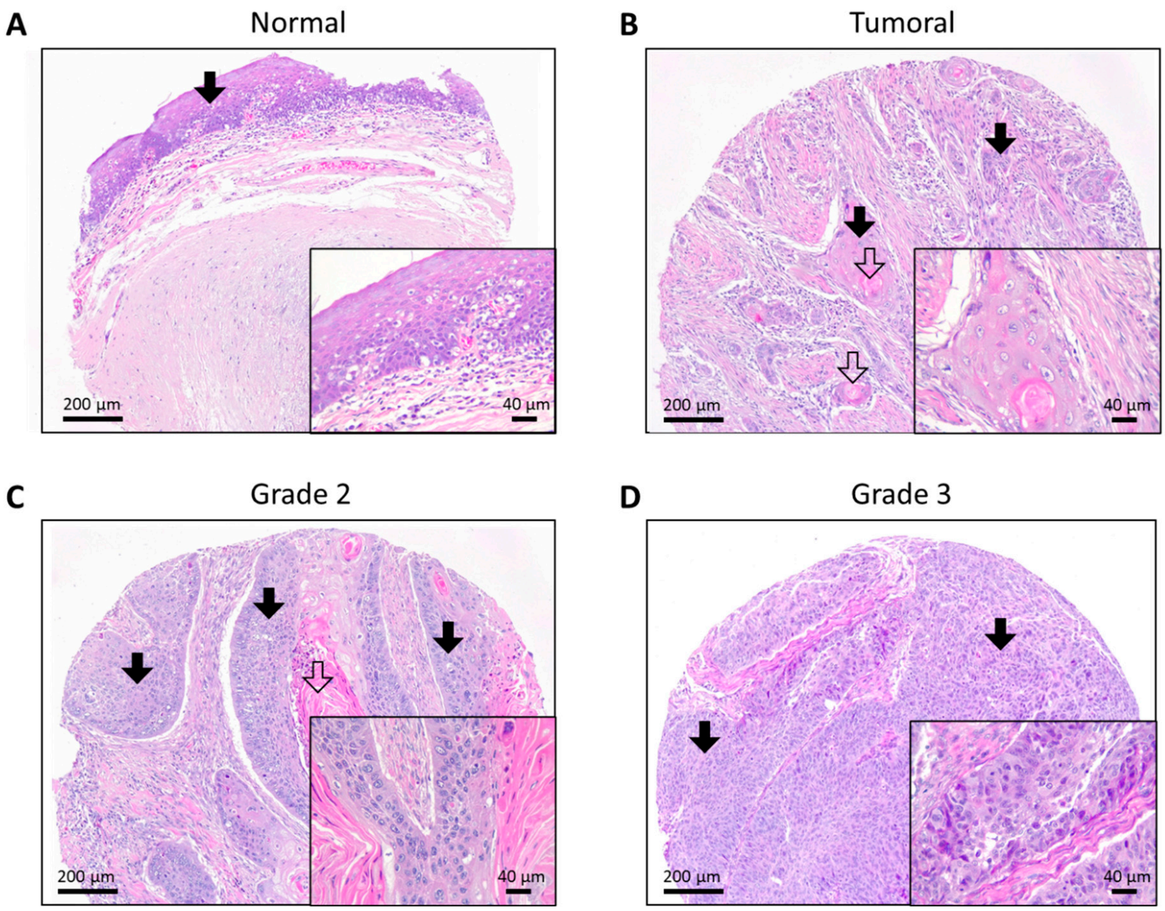

Figure S3. Hematoxylin and eosin-stained HNC human tissue sections. (A) Normal laryngeal epithelium corresponding to the mechanically exposed areas consists of an organized stratified squamous nonkeratinized epithelium (black arrow) with connective tissue and blood vessels. (Detail). Squamous epithelium detail and connective tissue with collagen fibers (pink color). (B). Groups of rounds epithelial cells (black arrow) lose the normal epithelial organization and form small dysplasia with squamous areas (empty arrow). Detail of rounded cells and squamous areas. (C). Big nodules of rounded cells (black arrows) with squamous areas (empty arrow) forms Dysplasia areas. (Detail). Nodule detail with rounded cells and squamous area. Black spots represent apoptotic and dividing cells. (D). High dysplasia grade with abnormal epithelial cell growth taking up most of the section (black arrow). (Detail) Dysplasia cells.

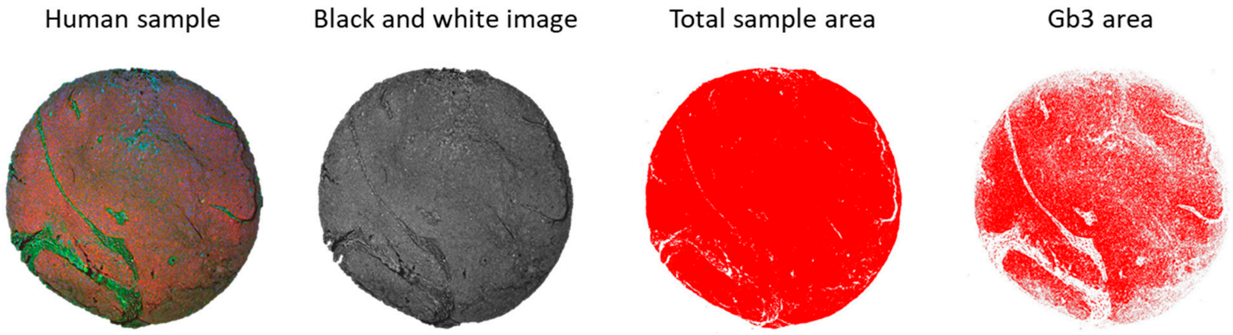

$$\text{Gb3 Area \%} = \frac{Gb3\ area\ x\ 100}{Total\ sample\ area}$$

Figure S4. GB3 quantification in human samples. The percentage of GB3 expression area was calculated considering the GB3 area versus the total area of the sample.
